# Micro CT evaluation of marginal discrepancies of endocrown restored molars with different intrapulpal depths and materials of fabrication. (in-vitro study)

**DOI:** 10.1186/s12903-025-05474-9

**Published:** 2025-01-27

**Authors:** Mohamed Aly Mohamed Badr Elagwany, Amina Mohamed Hamdy, Maged Mohamed Zohdy, Aliaa Mahrous, Ahmed Tawfik, Soha Osama Nabih

**Affiliations:** 1 Faculty of dentistry, Fayoum University, fayoum, Badr City, Cairo Egypt; 2 Faculty of dentistry, Ain Shams University, Cairo, Egypt; 3 Faculty of dentistry, MSA University, Giza, Egypt; 4 EPSRC Future Advanced Metrology Hub, University of Huddersfield, Huddersfield, UK

**Keywords:** Mandibular molars, Endocrowns, Marginal adaptation, Micro CT, Lithium disilicate, Polyetheretherketon (PEEK)

## Abstract

**Objective:**

The purpose of this study was to evaluate the effect of different preparation depths (0, 2 and 4 mm) of different restoration designs (classic endocrown design versus overlay design) on marginal adaptation of restorations fabricated of two different restorative materials (lithium disilicate and PEEK).

**Materials and methods:**

Sixty mandibular natural molars were collected as abutments for the restorations of this study, and grouped in three main groups of different cavity depths (0, 2 and 4). Each group was divided into two subgroups according to material of fabrication to (L) for lithium disilicate (IPS emax CAD, Ivoclar vivadent, Switzarland) and (P) for PEEK (Bio-hpp, Bredent, Germany). CAD/CAM milling technology was used for fabrication of restorations. After cementation of restorations over abutments, hydrothermal aging was performed, and then marginal adaptation was evaluated via micro CT technology.

**Results:**

Regarding cavity depths, there was a significant difference between different groups (*p* < 0.001). The highest value was found in samples with 4 mm extension (84.35 ± 18.16), followed by samples with 2 mm extension (66.52 ± 21.86), while the lowest value was found in samples without pulpal extension (59.41 ± 22.16). Post hoc pairwise comparisons showed samples with 4 mm extension to have a significantly higher value than samples without extension (*p* < 0.001). Regarding materials of fabrication, PEEK (85.32 ± 12.37) had a significantly higher value than Emax (54.86 ± 20.86) (*p* < 0.001).

**Conclusions:**

Increasing intrapulpal cavity depths increases vertical marginal gap of lithium disilicate or PEEK restorations. Endocrowns fabricated of lithium disilicate show less marginal discrepancies than that of endocrowns fabricated of PEEK.

## Introduction

Traditional ways for restoring endodontically treated teeth showed decreased clinical durability because of extensive weakening in the remaining tooth structure after endodontic treatment that increases the probability of root fracture under masticatory function [1, 2].

By release of new biomimetic and minimally invasive concepts, beside new adhesive strategies, computer aided designing / computer aided manufacturing and recent high strength restorative materials; new restoration designs have been introduced for rehabilitating endodontically treated dentition [3, 4]. 

Endocrown is a monolithic adhesive type of restoration that obtains its retention from pulp chamber and remaining tooth structure, with good coronal sealing and superior mechanical properties [5]. Friction with pulpal walls provides Macro-mechanical retention, whereas adhesive cementation provides micromechanical. Endocrown has Many advantages over conventional crowns such as ease of application, minimal clinical time, and proper aesthetic properties [6].

**Pissis** [7] was the introducer of pioneer technique of endocrown and has described it as the mono-block porcelain technique. **Bindle** and **Mörmann** [8] discriped it as adhesive endodontic porcelain crowns bonded to posterior teeth that had experienced endodontic treatment.

Endocrown preparation has no specific or defined design. 2 mm intra-radicular retentive feature was suggested by several studies to afford the sufficient retention and resistance features [9, 10], while other studies focused on the effect of the depth (shallow or deep depth) of this intra-pulpal cavity on the marginal and internal adaptation of the endocrown restorations [11, 12].

Cavity depth can influence endocrown retention and stability in function of internal cavity volume, cavity surface area, and marginal and internal discrepancy. severe attrition of the crown portion mandates shallow cavity depth preparation, while a deep cavity depth can be associated with less attrition on the coronal structure [12].

The exact dimensions of the central intra-pulpal retention cavity are not precisely determined, especially in cases of excessive loss of dental tissues, where only minimal intact tooth structure above the cemento-enamel junction is left so the need for further intra-radicular extension might be important demand. The incorporation of the available space inside the pulp chamber to the preparation design enhances the stability and retention of the restoration [13]. By increasing the depth of pulpal cavity thus increasing intracoronal extension provides greater surface area that can be utilized for adhesive retention and proper distribution of masticatory forces [14].

Many restorative materials had been prohibited for endocrown fabrication provided that it possess adhesive capacity such as etchable ceramics containing glass (Feldspathic, Leucite or Lithium disilicate based), resin composite, reinforced glass ceramics with Zirconia, hybrid resin nanoceramics or PEEK in order to adhesively bond to tooth structure.

Lithium disilicate glass ceramics are one of the most successful restorative materials used for the fabrication of different dental restorations because of its optimum esthetics, superior strength, and excellent adhesion properties to the tooth structures [15]. It’s composed of crystalline phase (approximately 70 vol %) which is incorporated in glassy matrix. In the partial crystallization process (so-called ‘blue’ state), platelet-shaped lithium meta-silicate crystals LiSiO3 (40 vol %) are formed and embedded in a glassy phase. It has flexural strength of 130 + 30 MPa, which makes the blocks easily milled. After that, tempering the milled restorations at 850 °C leads to formation of lithium disilicate crystals Li_2_O_2_Si_2_, so the milled restoration then has the final shade and flexural strength of 360 + 60MPa [16].

Polyetheretherketone (PEEK) is a synthetic type of polymer with proper biomechanical and inert chemical properties. It is reported that poly-aromatic semi-crystalline thermoplastic entity of Polyetheretherketone (PEEK) with its promising mechanical properties favor it for bio-medical applications.Using polyetheretherketone (PEEK) material for endocrowns fabrication was suggested because of its mechanical properties and superior biocompatibility. Due to its opaque white color, it requires veneering to improve the esthetics [17]. Nagi et al [18]. compared the marginal fit of both peek and lithium disilicate endocrowns and concluded that both restorationswere within the clinically acceptable range.

Because marginal adaptation affects periodontal condition in addition to acting as a barrier against microbial invasion, it is a critical criterion for the lifetime and efficacy of indirect restorations. A larger marginal gap may cause the luting material to be exposed to the oral environment more often, which could lead to its disintegration [19]. A weak cement seal invites bacterial infiltration, which causes the cement to deteriorate chemically and mechanically. This leads to repeated caries and damage to the pulp, which is crucial [20].

**McLean** [21] concluded that the clinically acceptable range of a marginal discrepancy is less than 120 μm in terms of longevity of the restoration after they examined more than 1000 crowns. **Kirsch C. et al.** [22] stated that marginal gap values of (8–206 μm) are acceptable. CAD/CAM manufacturers aim to produce restorations that provides marginal gap values of (25:40 μm) when cemented that is considered theoretically ideal.

Micro-computed tomography, also known as X-Ray Microscopy, or micro CT, is a scale of 3D X-ray imaging that has significantly improved resolution [23]. It truly embodies 3D microscopy, which is the non-destructive imaging of an object’s incredibly fine-scale internal structure, both in vivo and in vitro [24]. A variety of user-friendly desktop and floor-standing equipment are available for micro- and nano-tomography. With a resolution of sub-microns, it creates three-dimensional photographs of the interior microstructure and morphology of the sample [25, 26].

Due to variations in the electronic densities of the materials exposed to the X-ray beams, micro-CT has been used for nondestructive investigation of the marginal fit of indirect restorations. The acquired images enable the assessment of the adaptation line in two or three dimensions in a variety of cuts and directions, providing a large number of measurement sites and making the study of crucial distances easier [27–29]. In addition to offering the identification of crucial distances with a multitude of applications, micro-CT permits an adequate number of gap measurements. For scanning, the sample is positioned in the scanning tube such that it is perpendicular to the X-ray beam. Using image analysis software, measurements on the CT scan pictures can be obtained in both 2D and 3D [30].

So, the objective of this study was to evaluate the effect of different preparation depths (0, 2 and 4 mm) of different restoration designs (classic endocrown design versus overlay design) on marginal adaptation of restorations fabricated of two different restorative materials (lithium disilicate and PEEK), the null hypothesis stated that altering cavity depths or fabrication materials has no effect on the marginal adaptation of endocrown and overlay.

## Materials and methods

Materials are listed in(Table 1).

**Table 1:** Materials used in the study with its chemical

	Material	Brand	Chemical composition
**1**	Lithium disilicate. (Fig. 1).	IPS emax CAD Ivoclar vivadent Switzarland	57–80% Si02, 11–19% Li2O and other oxides Li2O, K2O, MgO, AL2O3, P2
**2**	Peek blank (Fig. 2).	bre.CAM Bio-hpp Bredent Germany	Repeating units of three phenyl rings, two ester groups and one keto group

### Sample size calculation

Sample size calculation was performed using G*Power statistical power analysis program(version 3.1.9.7) for sample size determination.A power analysis was designed to have adequate power to apply a statistical test of the null hypothesis that there is no difference would be found between tested groups. The minimal required sample size (n) was found to be (54) samples and this was sufficient to detect a large effect size(d) = 1.55 with an actual power (1- β error) of 0.8 (80%) and a significance level alpha (α) of (0.05) (5%) Sample size was increased to (60) samples to account for possible failures during testing.

### Samples selection and evaluation

Sixty caries free, recently extracted human mandibular first and second molar teeth were collected for the present study after having exemption from the research ethics committee of faculty of dentistry, Ain Shams University, Cairo, Egypt. This invitro study did not include any intervention with human participants or animals, however, written and oral concents were obtained from the patients to use their extracted teeth in this research. Teeth were extracted from uncontrolled diabetic patients and evaluated for intact and cracks-free surfaces. All external debris was removed with an ultrasonic scaler. A digital caliper was used to measure the dimensions of the teeth at cemento-enamel junction level with an average mean mesio-distal dimension 10.5 mm. ± 0.5 mm. and an average mean bucco-lingual dimension 9 mm. ± 0.5 mm. Any other discrepancied dimensions were excluded from the study.

Samples were stored under distilled water in separate closed container for each till the next steps. No endodontic treatment was done for all sample teeth according to a protocol advocated by **Yooseok S. et al.** [30] for purpose of standardization among samples.

Samples were randomly divided into three groups (0, 2, 4) (*n* = 20 each) by using a computer- generated list of random numbers (www.randomizer.org*)*. Each group samples recieved different intrapulpal cavity depths; 0, 2 and 4 mm depths, then each group was subdivided equally and randomly according to the restoration material into two subgroups (L) for lithium disilicate and (P) for PEEK (*n* = 10 each), all procedures performed by single operator.

### Samples preparation

**Fig. 1:**
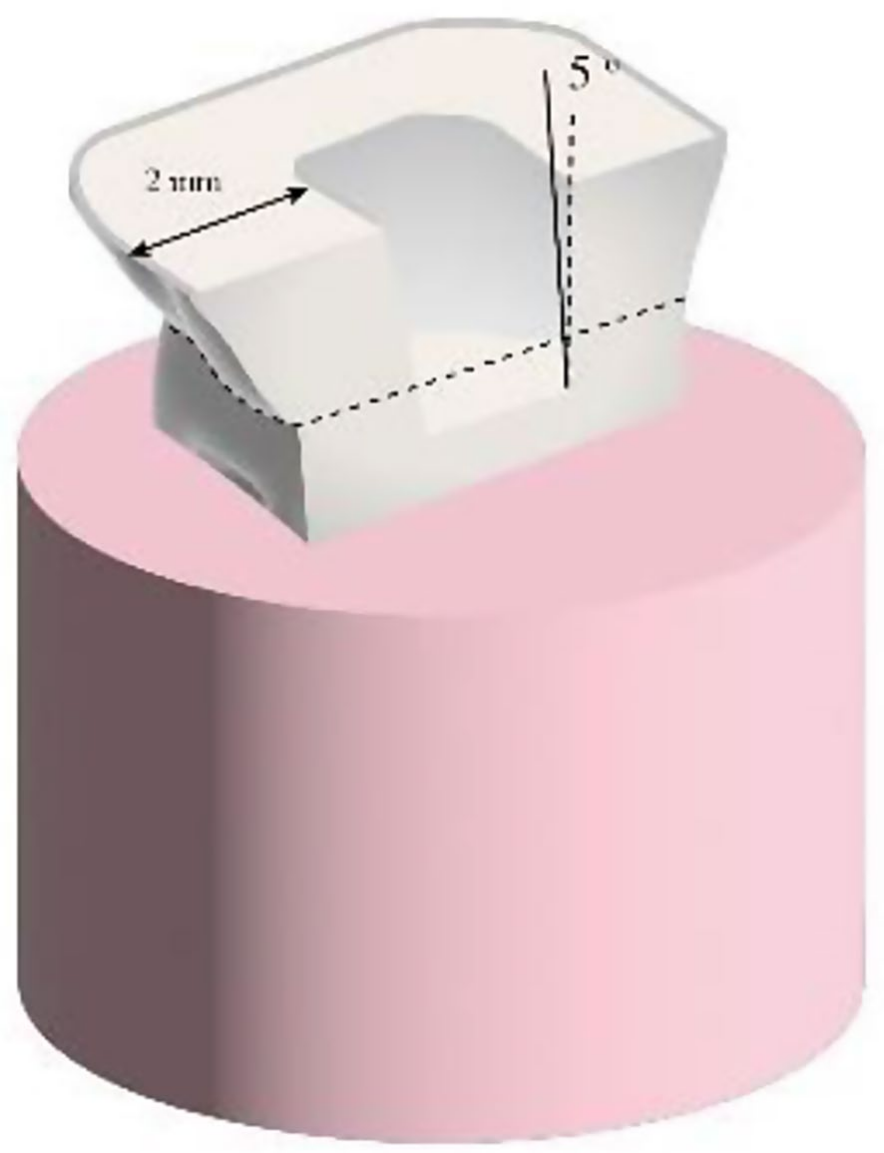
Schematic diagram showing 5 degrees axial wall divergence

Reduction of coronal portion was done 2 mm. from the cemento-enamel junction using diamond rotary wheel instrument (Intensiv wheel 817, Swisszerland) mounted on high speed headpiece leaving flat occlusal margin, and the margins were finished with butt joint preparation (meaning flat horizontal sidewalk prepration acts as finishing line without axial preparation). Pulpal cavity preparation was done with a tapered flat end diamond rotary instrument (Intensiv inlay kit, Swisszerland) mounted on high speed headpiece leaving a 5 degrees diverged axial walls with a total 10 degrees divergence. A 2 mm. circumferential occlusal band thickness; (two mm thickness of axial tooth structure) was created (Fig. 1). A paralleling device was used to mount handpiece for standardizing both flat butt reduction and occlusal divergence between samples.

### Preparation of different cavity depths

**Fig. 2:**
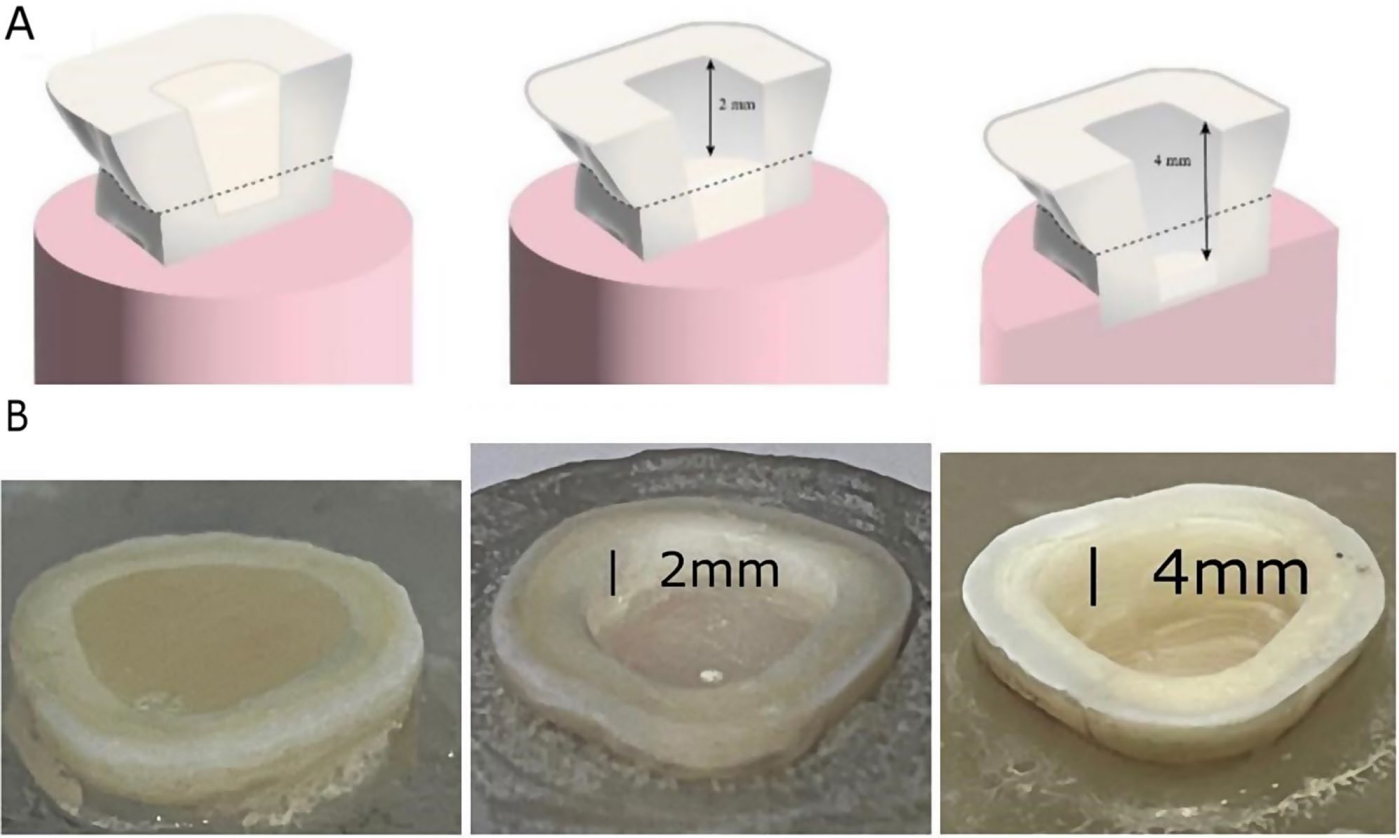
**A**) Schematic diagram showing different intrapulpal depths(0,2,4 mm), **B**)Prepared samples with different intrapulpal depths (0,2,4)mm

The preparation depths of all samples were adjusted using flowable composite according to their groups as follow: For group (0 depths) samples, obliteration of pulpal cavity to the level of occlusal margins was done leaving flat occlusal surface. For group (2 depths) samples, 2 mm pulpal cavity depth from the occlusal margins was left. For group (4 depths) samples (Fig. 2), 4 mm pulpal cavity depth from the occlusal margins was left [31, 32].

### Restorations fabrication

Optical scanning of all samples was done via extra oral scanner(DS mizar; egsolutions, Italy) with the following parameters: 2 × 5 megapixel cameras, accuracy (ISO 12836): 10 μm and scan speed (full arch): 30 s and then Digital scans of all samples were converted to STL format in order to create virtual dies using CAD system software; Exocad in-lab designing software(exocad GmbH, America, Inc). All 3D models were checked for detection of any undercuts or sharp point angles. Automatic margin line detection was used to trace the finish-line of all preparations.The luting space and adhesive gap were set at 50 μm.

**Fig. 3:**
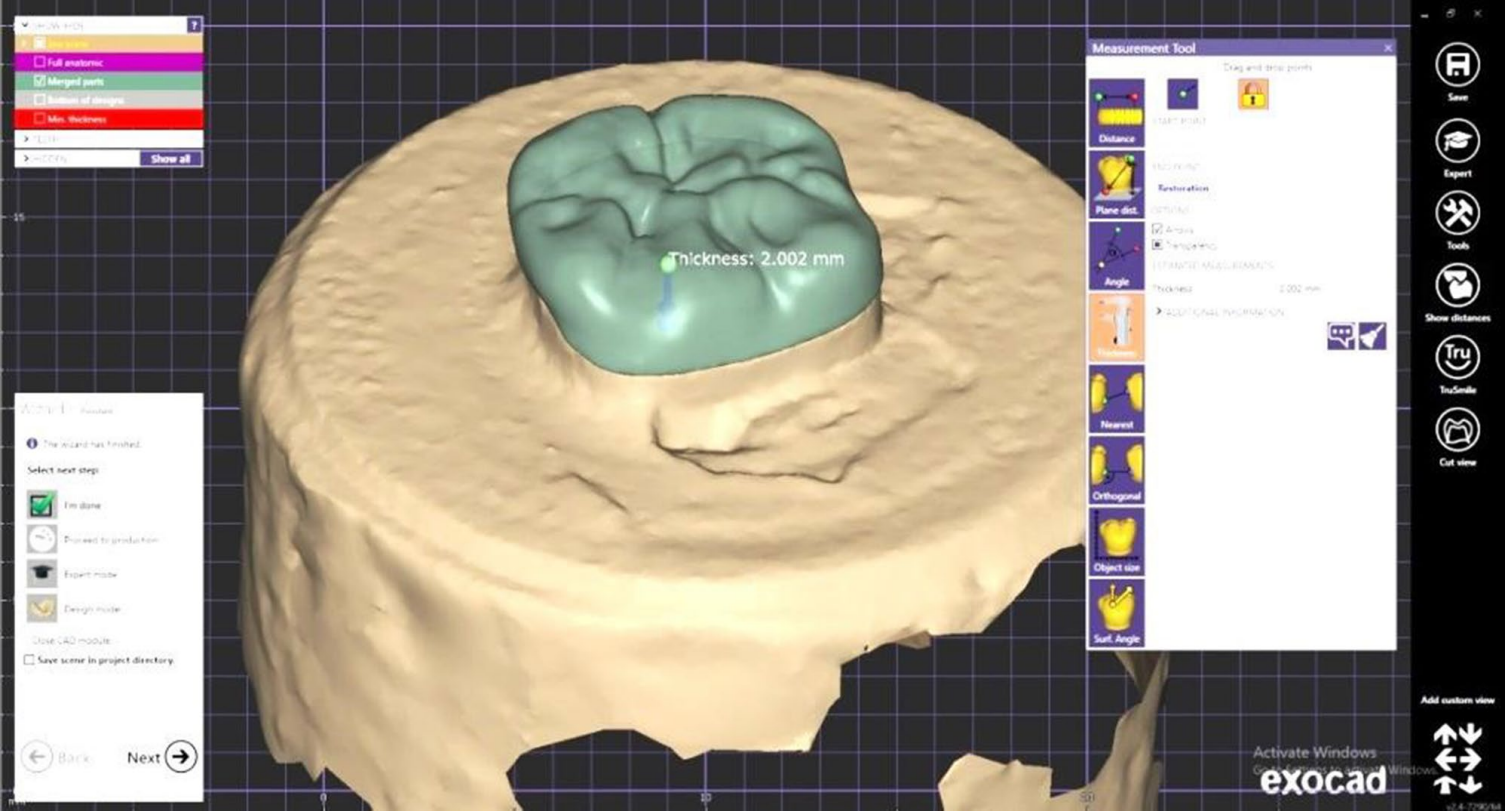
Original full anatomical design for fabrication of subgroup (L) restorations

**Fig. 4:**
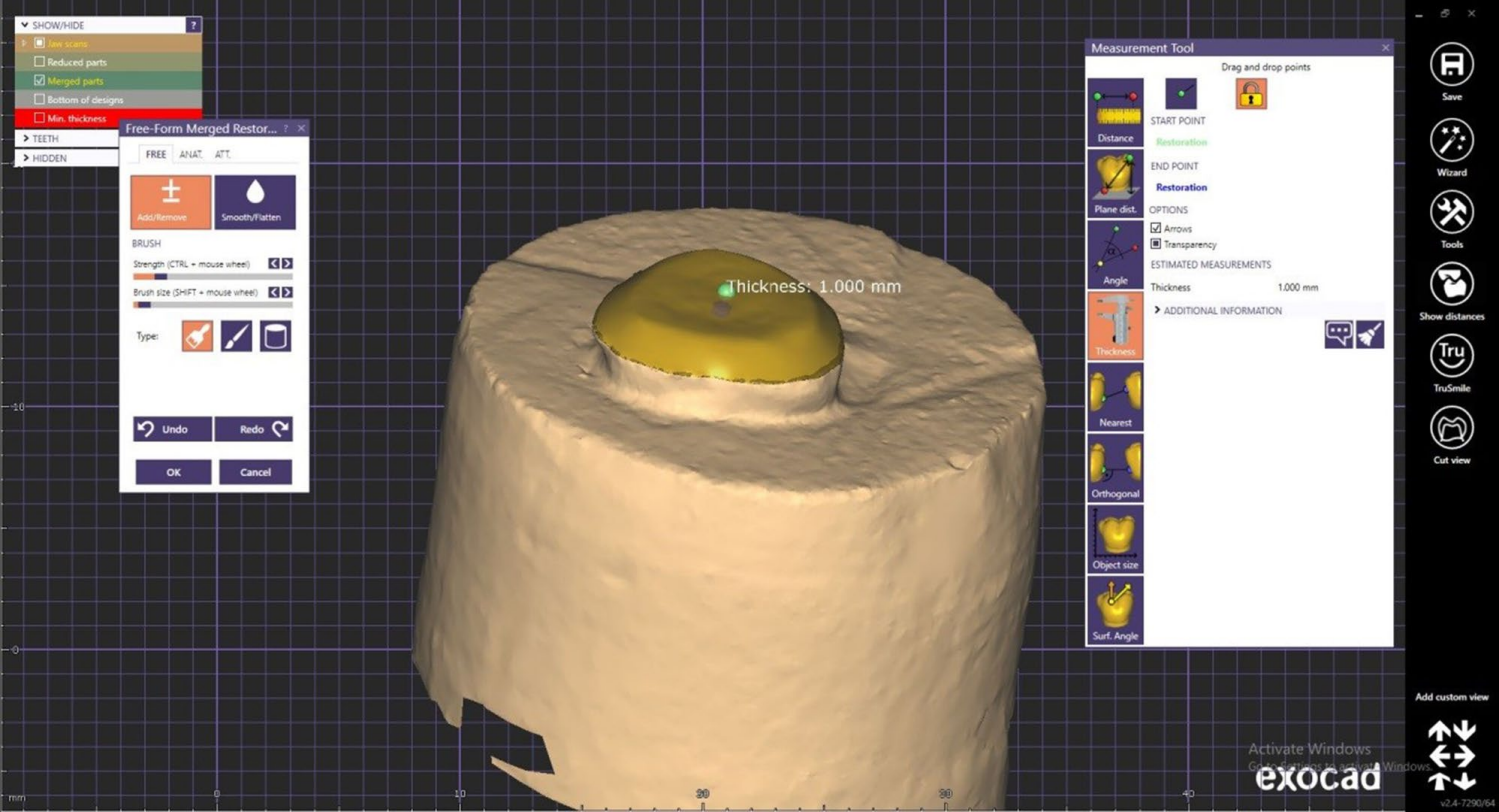
1 mm cutback for PEEK core fabrication

Regarding Lithium disilicate subgroup (L) a restoration of 2 mm. thicknesses was done, with anatomical occlusal aspect. The formed design was considered the final form and was ready for milling step (Fig. 3). For designing restoration of PEEK subgroup (P), a 1 mm. cutback of the original 2 mm thickness design was done leaving a room for composite veneer fabrication. (Fig. 4) So the final restoration would be 1 mm thickness PEEK core and 1 mm thickness composite veneer.

Milling of the restorations was done using SHERA Eco-Mill 5x; Bimedis, china milling machine. The milling preview was activated to start the milling process. The milling machine dialog box allows for choosing the type and size of the material according to the type of the restoration to be milled, then the block was fixed in the spindle of the milling machine and the door was closed then the milling icon was clicked to start the milling process.

**Fig. 5:**
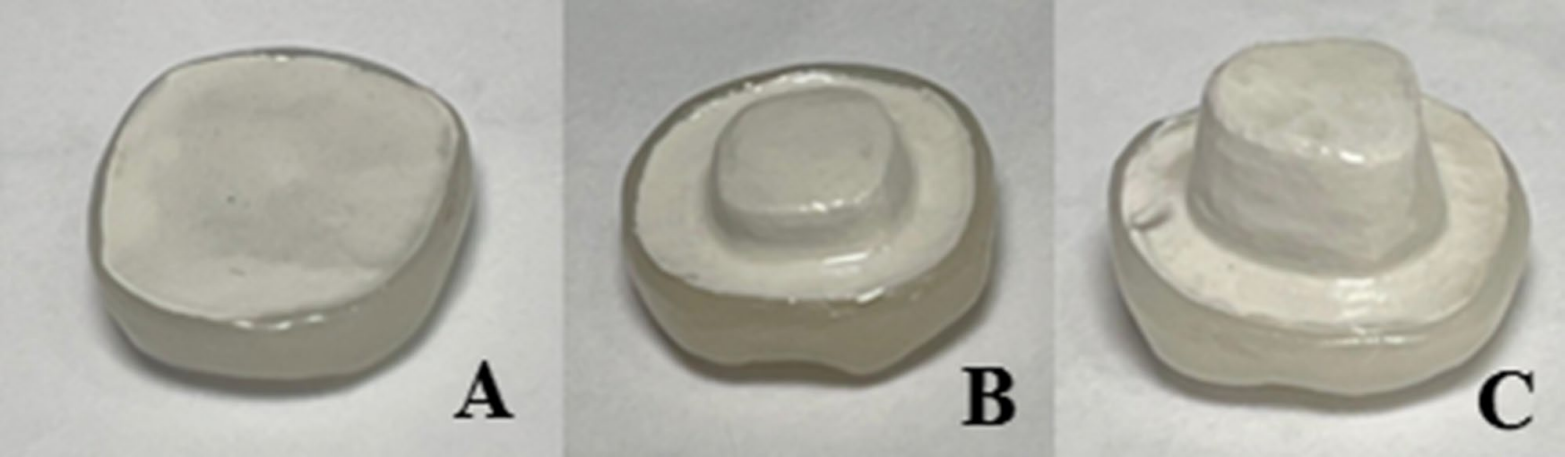
Cemented composite veneers over PEEK cores **A**) 0 mm depth **b**) 2 mm depth **c**) 4 mm depth

Regarding subgroup (L), the restorations were full anatomically milled using lithium disilicate ceramics blocks (IPS emax CAD, Ivoclar vivadent, Switzarland) of 2 mm thickness, then crystallization phase was adjusted at the furnace (programat P310). For subgroup (P), the restorations were fabricated as bi-layered structure *First*, the PEEK core of 1 mm thickness using Polyetheretherketone (Bio-hpp, Bredent, Germany). *Second*, indirect composite veneer of 1 mm thickness using composite resin veneering blank. Both components were connected with CAD-ON technique using dual cure composite resin after connected surfaces being sandblasted with 110 μm aluminum oxide grit at a pressure of 3 bars with 45 angle of application and 3 cm distance and after conditioning with light polymerized adhesive which then cured in a light polymerization unit according to manufacturer instructions. (Fig. 5)

### Restorations cementation

After restorations have been checked for marginal fit with inspection and dental explorer, cementation process was started with abutments acid etching with 37% phosphoric acid for 20 s on the enamel margin, then rinsed with water spray and dried with compressed air.

For subgroup (L), Acid etching of restorations with 9.5% hydrofluoric acid for 20 s was done, and then rinsed thoroughly for another 60 s then dried. Silane coupling agent was added and left to react for 60 s. For subgroup (P), sandblasting of the fitting surfaces was done with 110 μm aluminum oxide grit at a pressure of 3 bars with 45^o^ angle of application and 3 cm distance. Next, a universal MDP-containing adhesive (ALL-Bond Universal) was applied and light-polymerized for 20 s according to the manufacturer’s instructions.

Cementation was done using dual-polymerized self-adhesive resin cement at room temperature (Breeze) using the automix tip to the fitting surface of the restorations and inside the preparations. Cementation was done under constant loading via loading device with 1 kg pressure towards central fossae of the occlusal surface of the restorations, to minimize variation in pressure applied among samples during cementation.

### Aging

All samples were subjected to hydrothermal aging in autoclave at 134^o^ C and 2 MPa for five hours. This step was to mimic one year of oral conditions according to ISO standards 13,356 [33]

### Marginal gap measurement

**Fig. 6:**
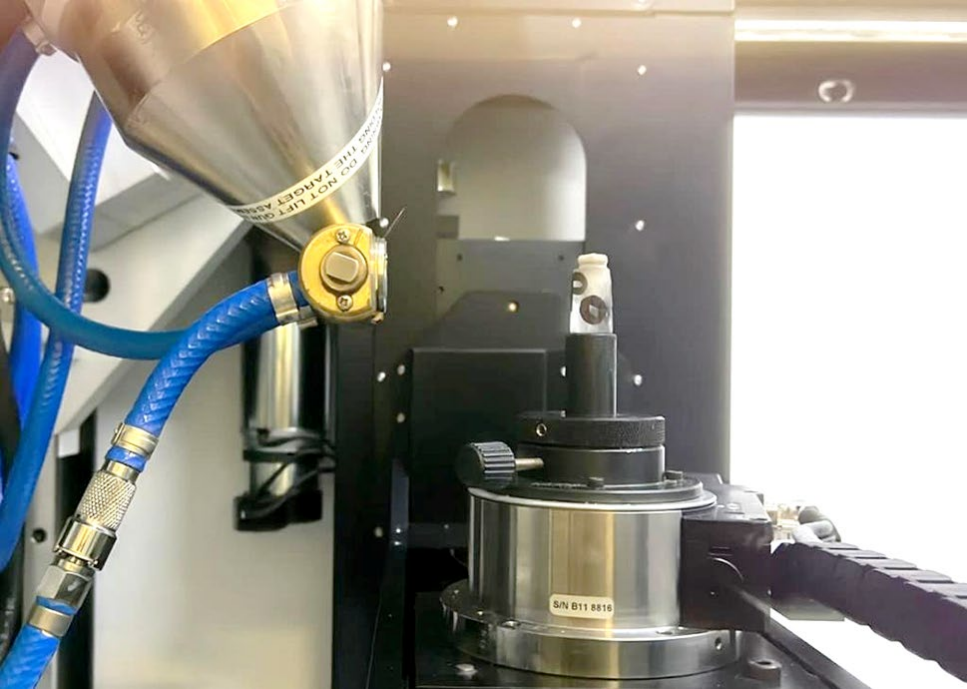
mounted sample for CBCT measurement

This phase was started with micro CT scanning of each sample, using Nikon XTH 225 ST system which was operated with the following parameters: 145 kV tube voltage, 145 µA tube current, and 1440 angular projections acquired over a rotation of 360◦, with an exposure time of 1.42 s. The software used is Inspect-X and the scanning session takes 70 min per tooth. (Fig. 6)

**Fig. 7:**
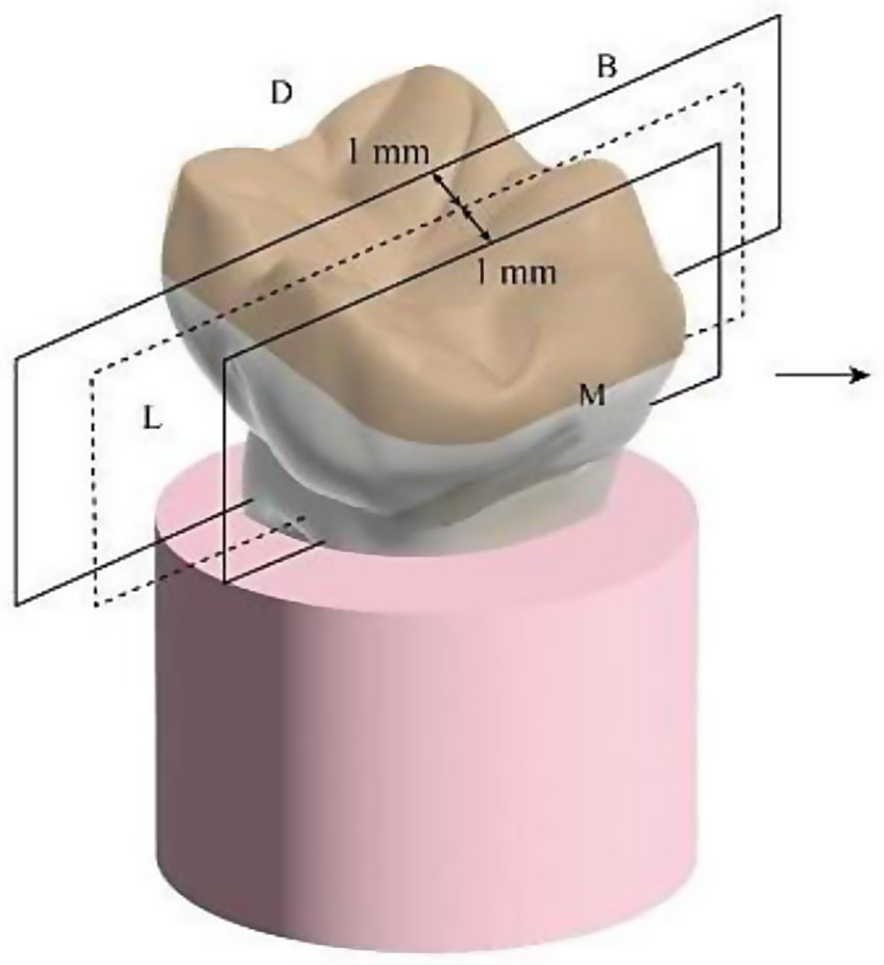
X axis (bucco-lingual cross sections)

**Fig. 8:**
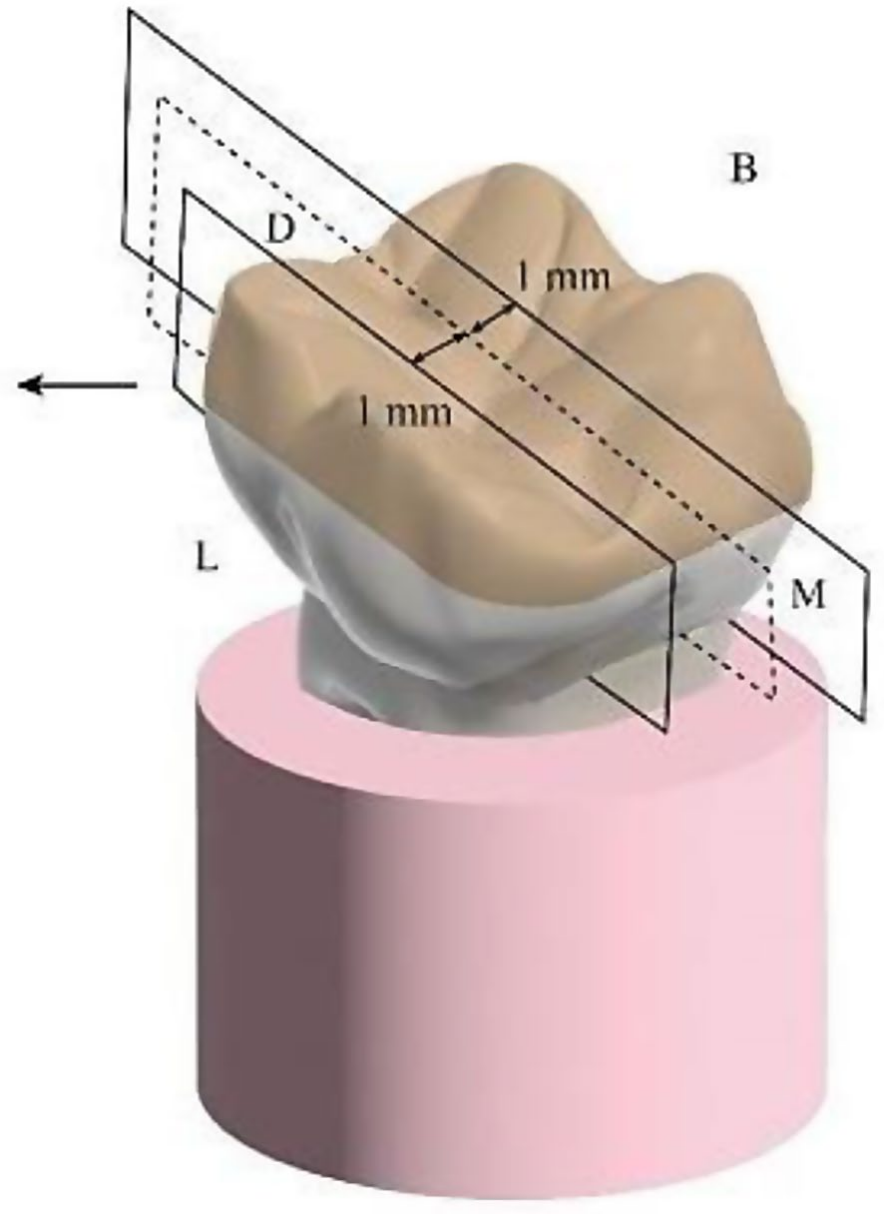
Y axis (mesio-distal cross sections)

**Fig. 9:**
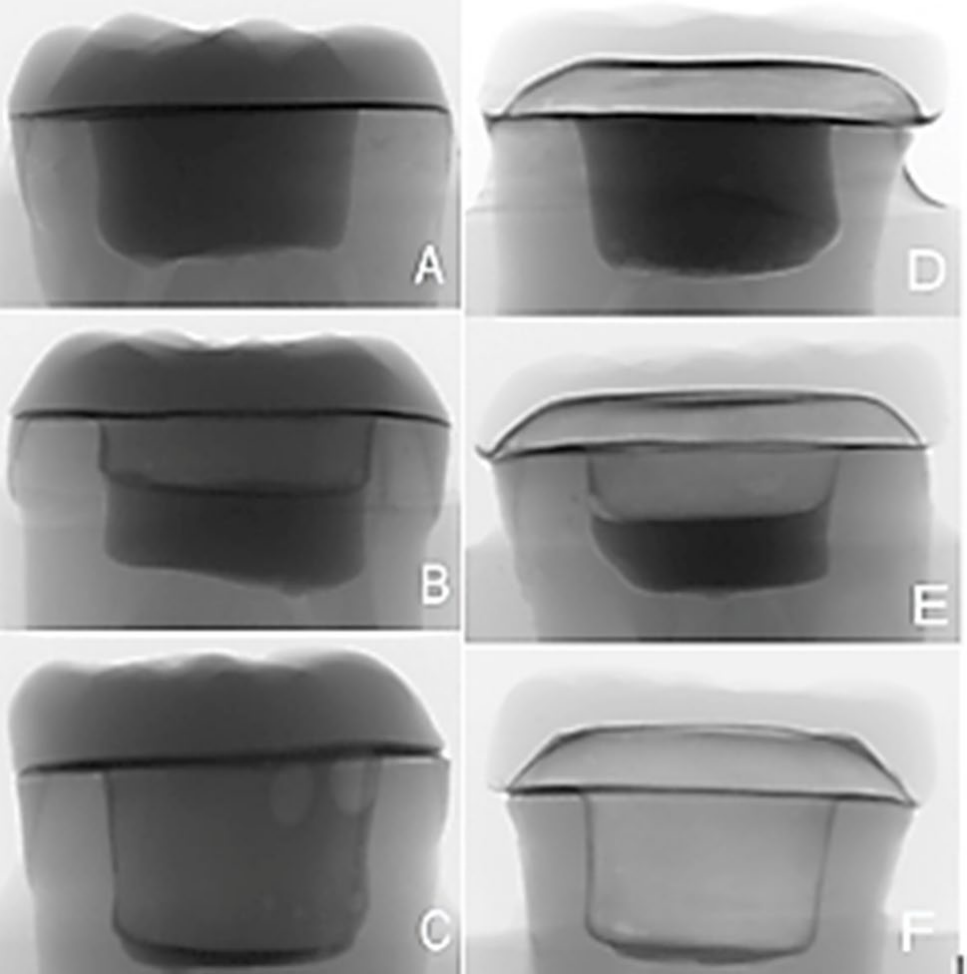
2D images of Constructed 3D design of each group; (**A**) L0, (**B**) L2, (**C**) L4, (**D**) P0, (**E**) P2 and (**F**) P4

The raw files were converted into bmp files using NRecon software, and CTAn was used for designing bmp CT files and reconstructing 3D images. For each design, six cross sections were obtained; three bucco-lingual at the X-axis (Fig. 7) and three mesio-distal at the Y-axis (Fig. 8). The distribution of cross sections at each axis (Y and X) was as follow: first cross section was through the center of the sample, and two additional cross sections were obtained bilaterally at 1 mm intervals. Six 2D Images were obtained from six cross sections of each constructed 3D design (three images from three cross sections of each axis). For calculating marginal gap of each 2D image, an average of six measurements at six reference points were obtained via 3D distance length tool value in microns (Fig. 17). To maintain a consistent position during these measurements, the pixel level in the X and Y axes were recorded using DataViewer software, and the µCT result analysis was carried out with a fixed gray value threshold ISO 50% for all scans and used for all specimens, by a single well-trained operator. (Fig. 9)

### Statistical analysis

Numerical data were presented as mean and standard deviation values. They were checked for normality using Shapiro-Wilk test. Data showed parametric distribution and were analyzed using two-way ANOVA followed by Tukey’s post hoc test. Comparison of main and simple effects was done utilizing Bonferroni correction with the pooled error term from the main ANOVA model. The significance level was set at *p* < 0.05 within all tests. Statistical analysis was performed with R statistical analysis software version 4.1.3 for Windows.

## Results

### Effect of different variables and their interaction

Effect of different variables and their interaction on marginal gap (µm) were presented in (Table 2).

**Table 2:** Effect of different variables and their interactions on marginal gap (µm) (µm)

Source	Sum of squares	df	Mean square	f-value	*p*-value
**Material**	9743.36	1	9743.36	60.02	< 0.001*
**Preparation design**	4623.02	2	2311.51	14.24	< 0.001*
Material * Preparation design	1298.33	2	649.17	4.00	0.027*

df = degree of freedom*; significant (*p* ≤ 0.05) ns; non-significant (*p* > 0.05)

There was a significant interaction between type of material and preparation design (*p* = 0.027).

### Main effects

#### A-Effect of material

Intergroup comparison, mean and standard deviation (SD) values of marginal gap (µm) for different materials (regardless the effect of preparation depths) were presented in (Table 3) showing that PEEK had a significantly higher value than Emax.

**Table 3:** Intergroup comparison, mean and standard deviation (SD) values of marginal gap (µm) for different materials

Marginal gap (µm) (mean ± SD)		*p*-value
Emax	PEEK	
54.86 ± 20.86	85.32 ± 12.37	< 0.001*

*; significant (*p* ≤ 0.05) ns; non-significant (*p* > 0.05)

#### B-Effect of preparation design

Intergroup comparison, mean and standard deviation (SD) values of marginal gap (µm) for different preparation designs (regardless the effect of materials) were presented in (Table 4) showing that there was a significant difference between different groups Post hoc pairwise comparisons showed samples with 4 mm extension to have a significantly higher value than samples without extension (*p* < 0.001).

**Table 4:** Intergroup comparison, mean and standard deviation (SD) values of marginal gap (µm) for different preparation designs

Marginal gap (µm) (mean ± SD)			*p*-value
0 mm	2 mm	4 mm	
59.41 ± 22.16^B^	66.52 ± 21.86^AB^	84.35 ± 18.16^A^	< 0.001*

Means with different superscript letters within the same horizontal row are significantly different *; significant (*p* ≤ 0.05) ns; non-significant (*p* > 0.05)

### Interactions

Intergroup comparisons, mean and standard deviation (SD) values of marginal gap (µm) for different materials and preparation designs were presented in (Table 5) and.

**Table 5:** Intergroup comparisons, mean and standard deviation (SD) values of marginal gap (µm) for different materials and preparation designs

Material	Marginal gap (µm) (mean ± SD)			*p*-value
	0 mm	2 mm	4 mm	
**Emax**	40.88 ± 10.27^B^	46.75 ± 5.90^B^	76.95 ± 20.89^A^	< 0.001*
**PEEK**	77.95 ± 12.55^A^	86.28 ± 9.46^A^	91.75 ± 12.26^A^	**0.105ns**
**p-value**	< 0.001*	< 0.001*	**0.132ns**	

Means with different superscript letters within the same horizontal row are significantly different *; significant (*p* ≤ 0.05) ns; non-significant (*p* > 0.05)

#### A-Effect of material with each preparation design

- 0 mm:

PEEK had a significantly higher value than Emax.

- 2 mm:

PEEK had a significantly higher value than Emax.

- 4 mm:

PEEK had a higher value than Emax yet the difference was not statistically significant.

#### B-Effect of preparation design within each material

- Emax:

There was a significant difference between different groups. The highest value was found in samples with 4 mm extension, followed by samples with 2 mm extension, while the lowest value was found in samples without extension. Post hoc pairwise comparisons showed samples with 4 mm extension to have a significantly higher value than other groups.

- PEEK:

There was no significant difference between different groups. The highest value was found in samples with 4 mm extension, followed by samples with 2 mm extension, while the lowest value was found in samples without extension.

## Discussion

The null hypothesis was rejected as there was a statistically signisicant difference between the values of test groups in marginal adaptation.

Endocrowns are a possible treatment alternative for the restoration of endodontically treated teeth with severe loss of coronal tooth tissue.Today, such restorations are most likely made of plenty of restorative materials, such as pressed glass ceramics, milled glass ceramics, hybrid ceramics or even composite blocks. Polyetheretherketone (PEEK) could be a possible alternative for ceramics [34, 35]. 

Good marginal adaptation of endocrown restored endo-treated dentition decreases microleakage considerably and the occurrence of secondary caries. It thus improves the longevity of the fillings [36]. The depth of pulpal cavity was proved to have effect on marginal discrepancies of endocrowns. Studies done by **Yooseok et al.** [30] **and El-damanhoury et al.** [12] proved that increasing inrapulpal cavity depth negatively affected the marginal adaptation.

The effect of material of fabrication on marginal adaptation of endocrown has been evaluated. **Godil et al.** [35] had used PEEK for fabrication of endocrown, and reported increased marginal gap compared to lithium disilicate.

The aim of this study was to evaluate the effect of different preparation depths (0, 2 and 4 mm) of different restoration designs (classic endocrown design versus overlay design) on marginal adaptation of restorations fabricated of two different restorative materials (lithium disilicate and PEEK).

Extracted human teeth were used in the present study to be similar to the clinical condition concerning the enamel and dentin bonding. In order to minimize the possible variations and to approach the desired standardization, selection of teeth of average sizes and almost similar shapes allowing maximum deviation of 10% from the determined mean was performed before testing [30]. This was done via selection of first and second mandibular molars with mean mesio-distal dimension of 10.5 mm. ± 0.5 mm. and mean bucco-lingual dimensions of 9 mm. ± 0.5 mm. which was measured by digital caliber.

Preparation was done via individual practitioned clinician for purpose of simulation of clinical situation, so as to anticipate representable results. Standard inlay preparation diamond kit was used via a high-speed hand-piece which was connected to paralleling holding device to make sure that there was fixed tapering of preparation among samples.

Butt margin design was used because it is considered the classical approach and almost all in-vitro studies were demonstrated using that design. butt joint designs provided a stable surface that resists the compressive stresses because it is prepared parallel to the occlusal plane [12].

Flowable composite was used as a base to modify cavity depth between groups, and it was selected because of low modulus of elasticity and coefficient of thermal expansion and contraction that was too close to that of dentin [32]. 

For PEEKs, bonding to PEEK is still quite challenging [37]. Airborne particle abrasion improved its micro-roughness [38], while pretreatment with methyl methacrylate-based (Visio.Link) adhesives increased wetting with the veneering material and demonstrated adequate chemical bond to PEEK [39].

In the present study, priming of intaglio surface of PEEK restoration was performed via universal bond containing mdp functional group. This was agreed with Chersoni S. et al. who found that water based SE bond is suitable for adhesion of hydrophobic and chemically inert surfaces, which quite caters to the properties of PEEK. The hydrophilic primer can penetrate into the porous surface of PEEK, thereby contributing to improved Shear Bond Strength [34]. 

In order to simulate the clinical conditions to which the restorations will be subjected, hydrothermal aging was carried out; All samples were subjected to autoclave at 134o C and 2 MPa for five hours, to mimic one year of oral conditions according to ISO standards 13,356 [33].

In this study, µCT was used to detect the marginal discrepancies after cementation, a technique that was conflicted by **Yooseok et al.** [30] protocol of measuring marginal discrepancies before cementation claiming that low radiographic contrast between cement and dentin would be confusing during analysis.

The results of this study revealed that all the tested samples showed mean values within the recommended clinical range (30.26 μm to 109.3 μm) as in the study by **McLean** [21] who has conducted a five-year study on over one thousand restorations and determined that 120 μm was the maximum acceptable marginal adaptation value.

This study revealed that endocrowns fabricated of lithium disilicate showed less marginal discrepancies (54.86 ± 20.86) than done endocrowns fabricated of PEEK (85.32 ± 12.37).This result was in agreement with **Godil et al.** [35] who found that endocrowns that were fabricated from lithium disilicate showed less marginal and internal discrepancies than those fabricated of PEEK, and they attributed their result due to the semi-crystalline structure of PEEK which contains fillers embedded in resin matrix, therefore creating differences in the milling of the two materials [40]. These differences were obvious in (Fig. 9) ) that showed irregular margins of subgroup P compared to more smooth milled restorations of subgroup L.

That wasn’t in agreement with **Hasanzade M. et al.** [41] who concluded that there was no significant differences in marginal adaptation between tested endocrowns fabricated of (Vita Enamic, IPS Emax CAD and Vita Suprinity).and the results were (71.00 ± 3176, 69.22 ± 23.49 and 77.52 ± 13.39) respectively.

A more obvious disagreement was observed by a study done be **Osman M. et al.** [39] which revealed a better marginal fit of PEEK endocrown versus endocrowns fabricated of Lithium disilicate. But theses confliction may be attributed to different method of endocrown fabrication, which was pressing instead of milling technique, beside the silicon replica method of measurement used in the mentioned study instead of micro-CT analysis used in the current study.

Cavity depth affected marginal adaptation of studied restorations. Increased cavity depth caused increased vertical discrepancies with 4 mm cavity depths of subgroup L (76.95 ± 20.89). But that increase in marginal discrepancies with increasing intra-pulpal cavity depth was not statistically significant for P subgroup with different cavity depths (0, 2 and 4) which where (77.95 ± 12.55, 86.28 ± 9.46 and 91.75 ± 12.26) respectively.

This outcome was confirmed by a conclusion of a previous studies by **Yooseok S. et al.** [30] and **Gaintantzopoulou et al.** [12]. They concluded that increased cavity depth of endocrown preparation increased vertical marginal gap. **Yooseok et al.** [30] evaluated the effect of cavity depths on internal and marginal adaptation of endocrowns and they attributed their findings to the fact that areas farther from the scanner is less accurate and undercuts couldn’t be easily detected which may interfere with restoration setting.

Another explanation of increased marginal discrepancy with increased cavity depth that increased axial length would increase surface area available for friction between fitting surface of restoration and cemented surface of the abutment, which interfere with seating of restoration compared to decreased axial length [42]. 

This study had some limitations as the endocrown and overlays were tested under invitro conditions which might be different from clinical conditions. further invivo studies are required.

## Conclusions

Within the limitations of this study, the following conclusions were got:

- Increasing intrapulpal cavity depths increases vertical marginal gap of restorations made of lithium disilicate or PEEK.
- Endocrowns fabricated of lithium disilicate show less marginal discrepancies than that of endocrowns fabricated of PEEK.

## Data Availability

The datasets used and/or analyzed during the current study are available from the corresponding authors on reasonable request.

## References

[1] Schestatsky R, Dartora G, Felberg R, Spazzin AO, Sarkis-Onofre R, Bacchi APG. Do endodontic retreatment techniques influence the fracture strength of endodontically treated teeth? A systematic review and meta-analysis. J Mech Behav Biomed Mater. 2019;90:306–12. 30396044 10.1016/j.jmbbm.2018.10.030

[2] Phang ZY, Quek SHQ, Teoh KH, Tan KBCTK. A retrospective study on the success, survival, and incidence of complications of post-retained restorations in premolars supporting fixed dental prostheses with a mean of 7 years in function. Int J Prosthodont. 2020;33(2):176–83. 32069342 10.11607/ijp.6090

[3] Sedrez-Porto JA, Rosa WL, da Silva AF. Munchow EA P-CT. Endocrown restorations: a systematic review and meta-analysis. J Dent. 2016;52:8–14. 27421989 10.1016/j.jdent.2016.07.005

[4] Govare NCME. Endocrowns: a systematic review. J Prosthet Dent. 2020;123(3):411–8. 31353111 10.1016/j.prosdent.2019.04.009

[5] Sedrez-Porto JA, Munchow EA, Cenci MS, P-CT. Which materials would account for a better mechanical behavior for direct endocrown restorations? J Mech Behav Biomed Mater. 2020;103:103–92. 10.1016/j.jmbbm.2019.103592 32090921

[6] Guo J, Wang Z, Li X, Sun C, Gao ELH. A comparison of the fracture resistances of endodontically treated mandibular premolars restored with endocrowns and glass fiber post-core retained conventional crowns. J Adv Prosthodont. 2016;8(6):489–93. 28018567 10.4047/jap.2016.8.6.489 PMC5179488

[7] of Fabrication P P. A metal-free ceramic restoration utilizing the monobloc technique. Pr Periodontics Aesthet Dent. 1995;7(5):83–94. 7548896

[8] Bindl AMW. Clinical evaluation of adhesively placed Cerec endo-crowns after 2 years–preliminary results. J Adhes Dent. 1999;1(3):255–65. 11725673

[9] Magne P, Carvalho AO, Bruzi G, Anderson RE, Maia HPGM. Influence of no ferrufe and no-Post buildup design on the fatigue resistance of endodontically treated molars restored with resin nanoceramic CAD/CAM crowns. Oper Dent 2014; 39595–602. 10.2341/13-004-L 25084102

[10] Forberger NGI. Influence of the type of post and core on in vitro marginal continuity, fracture resistance, and fracture mode of lithia disilicate-based all-ceramic crowns. J Prosthet Dent 2008; 100264–273. 10.1016/S0022-3913(08)60205-X 18922255

[11] Shin Y, Park S, Park JW, Kim KMPY. Evaluation of the marginal and internal discrepancies of CAD-CAM endocrowns with different cavity depths: an in vitro study. J Prosthet Dent. 2017;117:109–15. 27460311 10.1016/j.prosdent.2016.03.025

[12] HM. GMD and E-D. Effect of Preparation depth on the marginal and internal adaptation of computer-aided Design/Computer-assisted manufacture endocrowns. Oper Dent 2016; 41 607–16. 10.2341/15-146-L 27379835

[13] Biacchi GRBR. Comparison of fracture strength of endocrownsand glass fiber post-relained conventional crowns. Oper Dent. 2012;37(2):130–6. 21942234 10.2341/11-105-L

[14] Mörmann WH, Bindl A, Lüthy HRA. Effects of preparation and luting system on all-ceramic computer-generated crowns. Int J Prosthodont. 1998;11(4):333–9. 9758997

[15] Zoidis P, Bakiri EPG. Using modified polyetheretherketone (PEEK) as an alternative material for endocrown restorations: a short-term clinical report. J Prosthet Dent 2017,117335–339. 10.1016/j.prosdent.2016.08.009 27692583

[16] Aktas G, Yerlikaya HAK. Mechanical failure of endocrowns manufactured with different ceramic materials: an in vitro biomechanical study. J Prosthodont 2018;27340–346. 10.1111/jopr.12499 27465810

[17] Stawarczyk B, Thrun H, Eichberger M, Roos M, Edelhoff D, Schweiger J et al. Effect of different surface pretreatments and adhesives on the load-bearing capacity of veneered 3-unit PEEK FDPs. J Prosthet Dent 2015, 114666–673. 10.1016/j.prosdent.2015.06.006 26344191

[18] Nagi N, Fouda AM, Bourauel C. Comparative evaluation of internal fit and marginal gap of endocrowns using lithium disilicate and polyether ether ketone materials - an in vitro study Abstract BMC Oral Health 2023;23(1):207. 10.1186/s12903-023-02857-8 10.1186/s12903-023-02857-8 PMC10082505 37029396

[19] Dalloul RANJ. and A-HN. A comparative study of marginal fit between IPS e.max Press Crown and Endocrown after Cementation (in Vitro). Clin Med Diagn J 2016; 6 122 – 25.

[20] Laurent M, Scheer P, Dejou J. Clinical evaluation of the marginal fit of cast crowns—validation of the silicone replica method. J Oral Rehab. 2008;35(2):116–22. 10.1111/j.1365-2842.2003.01203.x 18197844

[21] JW M. The estimation of cement film thickness by an in vIvo technique. Br dent j 1971; 131107–111. 10.1038/sj.bdj.4802708 5283545

[22] Kirsch C, Ender A, Attin TMA. Trueness of four different milling procedures used in dental CAD/CAM systems. Clin O Investig. 2017;21(2):551–8. 10.1007/s00784-016-1916-y 27469100

[23] Kim JH, Jeong JH, Lee JHCH. Fit of lithium disilicate crowns fabricated from conventional and digital impressions assessed with micro-CT. J Prosthet dent. 2016;116(4):551–7. 27422237 10.1016/j.prosdent.2016.03.028

[24] Pelekanos S, Koumanou M, Koutayas SO, Zinelis S. Micro-CT evaluation of the marginal fit of different In-Ceram alumina copings. Eur J Esthet Dent. 2009;4(3):278–92. 19704928

[25] Borba M, Miranda WG Jr., Cesar PF, Griggs JA. Evaluation of the adaptation of zirconia-based fixed partial dentures using micro-CT technology. Braz O Res. 2013;27(5):396–402. 10.1590/S1806-83242013000500003 24036977

[26] Gielkens P, de Schortinghuis J. A comparison of micro-CT, microradiography and histomorphometry in bone research. Arch Oral Biol. 2008;53(6):558–66. 18190892 10.1016/j.archoralbio.2007.11.011

[27] Neves FD, Prado CJ. Micro-computed tomography evaluation of marginal fit of lithium disilicate crowns fabricated by using chairside CAD/CAM systems or the heat-pressing technique. J Prosthet Dent. 2014;112(5):1134–40. 24969409 10.1016/j.prosdent.2014.04.028

[28] Seo D, Yi YRB. The effect of preparation designs on the marginal and internal gaps in CEREC3 partial ceramic crowns. J Dent. 2009;37:374–82. 19282083 10.1016/j.jdent.2009.01.008

[29] Habib SR, Asiri W. Effect of anatomic, semi anatomic and non-anatomic occlusal surface tooth preparations on the adaptation of zirconia copings. J Adv Prosthodont. 2014;6(6):444–50. 25551003 10.4047/jap.2014.6.6.444 PMC4279041

[30] Yooseok Shin S, Park J-W, Park -Mahn, Kim Y-B, Park. and B-DR. Evaluation of the marginal and internal discrepancies of CAD-CAM endocrowns with different cavity depths: an in vitro study. J Prosthet Dent 2017 117(1) 109–15. 10.1016/j.prosdent.2016.03.025 27460311

[31] Manhart J, Chen H, Hamm GHRBML. Review of the clinical survival of direct and indirect restorations in posterior teeth of the permanent dentition. Oper Dent. 2004;29(5):481–506. 15470871

[32] Demarco FF, Correa MB, Cenci MS, Moraes ARON. Longevity of posterior composite restorations: not only a matter of materials. Dent Mater. 2012;28(1):87–101. 22192253 10.1016/j.dental.2011.09.003

[33] (2008) 109. ISO-Standerd. ISO 13356 Dental materials:implants for surgery-ceramic materials based on ytrria-stabilized tetragonal zirconia (Y-TZP): Geneve. Int Organ Stand.

[34] Chuang SF, Chang CH, Yaman PCLT. Influence of enamel wetness on resin composite restorations using various dentine bonding agents: part I - effects on marginal quality and enamel microcrack formation. J Dent. 2005;33(1):1–9. 16169654 10.1016/j.jdent.2005.07.006

[35] Godil AZ, Kazi AI, Wadwan SA, Gandhi KY, Dugal RJS. Comparative evaluation of marginal and internal fit of endocrowns using lithium disilicate and polyetheretherketone computer-aided design - computer-aided manufacturing (CAD-CAM) materials: an in vitro study. J Conserv dent. 2021;24(2):190. 34759588 10.4103/JCD.JCD_547_20 PMC8562835

[36] OH K, DH K. SH P. The influence of elastic modulus of base material on the marginal adaptation of direct composite restoration. Oper dent 2010; 35441–447. 10.2341/09-372-L 20672729

[37] Meshreky M, C H. H. K. Vertical marginal gap distance of CAD/CAM milled BioHPP PEEK coping veneered by HIPC compared to zirconia coping veneered by CAD-On lithium disilicate in-vitro study. Adv Dent J 2020;243–50.

[38] Stawarczyk B, Jordan P, Schmidlin PR, Roos M, Eichberger M, Gernet W et al. PEEK surface treatment effects on tensile bond strength to veneering resins. J Prosthet Dent 2014;1121278–1288. 10.1016/j.prosdent.2014.05.014 24969411

[39] Osman AM, Mahallawi E, Khair-Allah OS, L. S., Khodary E. NA. Marginal integrity and clinical evaluation of polyetheretherketone (PEEK) versus lithium disilicate (E-Max) endocrowns: Randomized controlled clinical trial. Int J Heal Sci 6(s4), 1831–45.

[40] Wagner C, Stock V, Merk S, Schmidlin PR, Roos M, Eichberger M et al. Retention load of telescopic crowns with different taper angles between cobalt-chromium and polyetheretherketone made with three different manufacturing processes examined by pull-off test. J Prosthodont 2018;27162–168. 10.1111/jopr.12482 27037795

[41] Hasanzade M, Sahebi M, Zarrati S, Payaminia LAM. Comparative evaluation of the internal and marginal adaptations of CAD/CAM endocrowns and crowns fabricated from three different materials. Int J Prosthodont 2020 Dec 19. 10.11607/ijp.6389 31856266

[42] Nakamura T, Dei N, Kojima TWK. Marginal and internal fit of Cerec 3 CAD/CAM all-ceramic crowns. Int J Prosthodont 2003; 116. 12854786

